# Atherosclerosis indexes and incident T2DM in middle-aged and older adults: evidence from a cohort study

**DOI:** 10.1186/s13098-023-00992-4

**Published:** 2023-02-17

**Authors:** Xin Wu, Yu Gao, Miyuan Wang, Hongye Peng, Di Zhang, Biyuan Qin, Liang Pan, Guolong Zhu

**Affiliations:** 1 Affiliated Shenzhen Maternity & Child Healthcare Hospital, Southern Medical University, Shenzhen, 518028 China; 2 College of Chinese Medicine, Beijing University of Chinese Medicine, Beijing, 100029 China; 3 School of Public Health, Huazhong University of Science and Technology, Wuhan, 430074 China; 4 Graduate School, Beijing University of Chinese Medicine, Beijing, 100029 China; 5 Chengdu University of Traditional Chinese Medicine, Sichuan, 610075 China; 6 Department of Science & Education, Deyang People’s Hospital, Sichuan, 618000 China; 7 Phase 1 Clinical Trial Center, Deyang People’s Hospital, Sichuan, 618000 China; 8 College of Traditional Chinese Medicine, Three Gorges University & Yichang Hospital of Traditional Chinese Medicine, Hubei, 443003 China

**Keywords:** Type 2 diabetes mellitus, Insulin resistance, Cohort study, Lipid comprehensive index, Atherosclerosis indexes

## Abstract

**Background:**

Type 2 diabetes mellitus (T2DM) is an expanding global health problem, requiring effective methods for predicting and diagnosing in its early stages of development. Previous studies reported the prognostic value of the atherosclerosis indexes in both cardiovascular diseases and T2DM. However, the predictive performance of Non-HDL-C, AI, AIP, TG/HDL-C and LCI indexes on the risk of T2DM remains unclear. This study aims to compare the five atherosclerosis indexes for predicting T2DM in middle-aged and elderly Chinese.

**Methods:**

Data are collected from wave 2011 and wave 2015 of China Health and Retirement Longitudinal Study (CHARLS). Multi-variate logistic regression models were used to estimate odds ratio (OR) with 95% confidence interval (CI) of incident T2DM with five atherosclerosis indexes, and the restricted cubic splines were used to visualize the dose–response relationships. Receiver operating characteristic (ROC) curve was drawn and the areas under the curve (AUC) were used to compare the performance of the five atherosclerosis indexes in predicting T2DM.

**Results:**

A total of 504 (10.97%) participants had T2DM. Multi-variate logistic regression analysis showed that five atherosclerosis indexes were associated with T2DM, with adjusted ORs (95% CIs) of 1.29 (1.15–1.45), 1.29 (1.18–1.42), 1.45 (1.29–1.62), 1.41 (1.25–1.59) and 1.34 (1.23–1.48) for each IQR increment in Non-HDL-C, TG/HDLC, AI, AIP and LCI, respectively. Restricted cubic spline regression showed a nonlinear correlation between five atherosclerosis indexes and the risk of T2DM (p for nonlinear < 0.001). According to the ROC curve analysis, LCI had the highest AUC (0.587 [0.574–0.600]).

**Conclusion:**

We found that LCI, compared with other indexes, was a better predictor in the clinical setting for identifying individuals with T2DM in middle-aged and elderly Chinese. LCI monitoring might help in the early identification of individuals at high risk of T2DM.

## Introduction

Diabetes is a serious and increasing global health burden, especially in developing countries [1]. With its large population, Asia is a major area of the rapidly emerging T2DM global epidemic. And the prevalence of T2DM in China drastically increased in the past few decades [2]. It is crucial to develop effective methods to predict and diagnose T2DM more accurately at its early stages.

Type 2 diabetes mellitus is recognized as a condition of complexity and heterogeneity [3]. Patients with T2DM typically have hyperglycemic metabolic abnormalities, and the major goal of diabetes care is to normalize blood glucose levels [4, 5]. Additionally, T2DM patients commonly have hypertension and dyslipidemia, which are recognized risk factors for atherosclerotic cardiovascular diseases (ASCVDs) [6]. Patients with hyperlipidemia often develop diabetes because they have increased intake of triglycerides and free fatty acid, which are deposited in internal organs and often induce insulin resistance (IR), leading to diabetes if it persists [7]. In addition, the presence of atherogenic dyslipidemia in patients with T2DM is often associated with hyperglycemia, as well as decreased HDL particles and LDL levels overlapping those of non-diabetics. Nevertheless, they also present small and dense LDL particles that are highly atherogenic and significantly increase the cardiovascular risk of these patients [8]. Hyperglycemia may cause a decrease in LDL receptors, resulting in a reduction in lipoprotein uptake and metabolism [9]. So diabetes and hyperlipidemia have mutual effect on each other.

The atherogenic index of plasma (AIP), a logarithmically transformed ratio of triglyceride (TG) to high-density lipoprotein-cholesterol (HDL-C) in molar concentration, was reported to be a sensitive marker of lipoprotein profiles [10]. Previous studies have shown that high lipid parameters, especially AIP, were risk factors of T2DM [11]. However, to date, no study has systematically evaluated and compared the performance of Non-HDL-C, AI, AIP, TG/HDL-C and lipid comprehensive index (LCI) in predicting the risk to T2DM.

Based on a sizable prospective cohort, this study aims to assess the value of Non-HDL-C, AI, AIP, TG/HDL-C and LCI indexes for T2DM prediction Chinese people aged 45 and older.

## Method

### Study population

Data were collected from the 2011 and 2015 waves of the CHARLS survey, which was freely accessible to general public (http://charls.pku.edu.cn/). By the year of 2015, the survey includes 21,097 residents and 12,241 households. CHARLS is a national-scale, interdisciplinary study that surveys residents aged 45 and above from 450 villages and communities across 28 provinces. In addition to information about personal characteristic, CHARLS offers information on family, health status, cognition function, retirement and personal property status. The database provides reliable information regarding health conditions and related effective features in middle-aged and older people.

The venous blood samples were collected by trained staff after participants had fasted for at least 12 h overnight in 2011 and 2015. On site, complete blood count was conducted immediately. Blood specimens were held at 4 degrees Celsius for further analysis, and all other samples were sent to a central laboratory located in Beijing. The levels of glucose, TC, TG, LDL-C and HDL-C were determined by enzymatic-colourimetric assay. Glycated haemoglobin levels were determined by high performance liquid chromatography (HPLC) with boronate affinities.

According to the purpose of this study, we settled on the study participants' eligibility requirements: aged 45 and older; detailed demographic information, including location, marriage, education, and gender; entire collection of fasting blood. Finally, A total of 4,596 participants from CHARLS were included. The CHARLS survey project was approved by the Biomedical Ethics Committee of Peking University, and all participants were required to sign informed consent.

## Measurement

### Assessment of five atherosclerosis indexes

We assessed atherosclerotic indexes at baseline such as Non-HDL-C, AI, AIP, TG/HDL-C and LCI and calculated them by the following formulas:

1. Non-HDL-C = TC-HDL-C
2. AI = (TC-HDL-C)/HDL-C
3. AIP = log^(TG/HDL−C)^
4. TG/HDL-C = TG/HDL-C
5. LCI = TC*TG*LDL-C/HDL-C

### Assessment of T2DM

According to the American Diabetes Association's 2005 definition [12], T2DM is defined by: a fasting blood sugar of 126 mg/dL (7 mmol/L) or higher, and/or a random blood sugar of 200 mg/dL (11.1 mmol/L) or higher, and/or a 6.5% HbA1c level or higher, and/or self-reported diabetes/hyperglycemia history. (“Have you ever had a diabetes or hyperglycemia diagnosis?”).

### Assessment of covariates

Analysis was adjusted for socio-demographic characteristics, health concerning behaviors and anthropometric measurements. The demographic variables analyzed were age, gender, education level (elementary school and below, high school, college and higher), location (urban, rural) and marriage (married, never-married/separated/widowed). For health concerning behaviors, we examined smoking habits (never, former smoker, present smoker), drinking habits (never, drink less than once a month, drink more than once a month) and sleep time. Those data were obtained by trained interviewers from self-reported files. Anthropometric measurements include systolic blood pressure and diastolic blood pressure, which were the mean of the three-time measurements using sphygmomanometer HEM-7200 from Omron.

### Statistical analysis

For normally distributed data, means and standard deviation were used. For non-normally distributed data, medians (interquartile range, IQR) were used. Percentages were used to describe classified variables. Each quartile (Q1, Q2, Q3, Q4) of the five atherosclerosis indexes' baseline characteristics and incident diabetes were compared with chi-square, Kruskal–Wallis H and ANOVA tests. Logistic model was used to estimate the odds ratio and 95% confidence interval of T2DM using those indexes as both categorical variables (quartile) and continuous variables (IQR increase). We used three models to determine the link between those five indexes and incident T2DM: a Model 1 as an unadjusted rough model; a Model 2 adjusted for education, sex, age, location and marriage; a Model 3 adjusted for sleep duration, drinking, and smoking. Restricted cubic splines (RCS) were used to determine any non-liner association between those five indexes and incident T2DM, as well as their dose–response relation. In order to assess the predicting effectiveness of the Non-HDL-C, TG/HDL-C, AI, AIP, and LCI for T2DM, we employed receiver operating characteristic (ROC) curve and the area under the curve (AUC) value. The AUCs between LCI and other indexes were compared using DeLong et al.’s non-parametric method [13].The best cutoff values of T2DM prediction were determined according to the Youden index.

R 4.1 was used to complete the statistical analyses. And the the "rms" package was used to complete restricted cubic splines. The significance test was performed using MedCalc version 13.0 for Windows (MedCalc Software, Mariakerke, Belgium) to compare the AUCs. Statistical significance for a two-tailed test was defined as *P* < 0.05.

## Result

### Baseline characteristics

Characteristics of the study participants were presented in Table 1. In all, 4,596 participants (median age = 58, with 2,152 (46.82%) men and 2,444 (53.18%) women)) were included, of which 504 (10.97%) had T2DM. The baseline medians (IQR) of Non-HDL-C, TG/HDL-C, AI, AIP and LCI in all participants were 134.92 (112.89, 158.51), 1.93 (1.24, 3.07), 2.66 (2.01, 3.48), 0.29 (0.09, 0.49), 41,471.98 (23,697.46, 73,052.4), respectively. Compared to participants without T2DM, those with T2DM presented obviously different characteristics, the latter were probably to have higher Glucose level, higher BMI/SBP/DBP/TG/WC, higher levels of L-DLC, Non-HDL-C, TG/HDL-C, AI, AIP, LCI, lower HDL-C levels, and be older.

**Table 1:** Baseline characteristics of participants with/without T2DM (N = 4,596)

	Total (n = 4596)	Non-T2DM (n = 4092)	T2DM (n = 504)	*p*
Age	58 (52, 65)	58 (51, 64)	60 (53, 67)	< 0.01
Gender				0.87
Female	2926 (53.34)	2594 (53.29)	332 (53.72)	
Male	2560 (46.66)	2274 (46.71)	286 (46.28)	
Marita				0.01
Married	4881 (88.97)	4350 (89.36)	531 (85.92)	
Non-Married	605 (11.03)	518 (10.64)	87 (14.08)	
Education				0.07
Elementary school and below	3866 (70.47)	3408 (70.01)	458 (74.11)	
High school	1127 (20.54)	1010 (20.75)	117 (18.93)	
College and higher	493 (8.99)	450 (9.24)	43 (6.96)	
Location				0.45
Urban	5142 (93.73)	4558 (93.63)	584 (94.5)	
Rural	344 (6.27)	310 (6.37)	34 (5.5)	
Smoking				0.73
Never	3344 (60.96)	2976 (61.13)	368 (59.55)	
Former smoker	423 (7.71)	375 (7.7)	48 (7.77)	
Present smoker	1719 (31.33)	1517 (31.16)	202 (32.69)	
Drinking				0.04
Never	3638 (66.31)	3201 (65.76)	437 (70.71)	
Drink less than once a month	453 (8.26)	412 (8.46)	41 (6.63)	
Drink more than once a month	1395 (25.43)	1255 (25.78)	140 (22.65)	
Sleep time	6 (5, 8)	6 (5, 8)	6 (5, 8)	0.74
BMI (kg/m^2^)	22.9 (20.73, 25.35)	22.78 (20.65, 25.17)	23.87 (21.38, 26.68)	< 0.01
WC (cm)	83.55 (77, 90.3)	83 (77, 90)	87 (79.2, 94)	< 0.01
Glucose (mg/dl)	99.9 (93.24, 106.92)	99.36 (92.88, 106.2)	104.31 (96.35, 111.6)	< 0.01
TC (mg/dl)	187.89 (165.85, 211.08)	187.5 (165.46, 210.41)	192.91 (170.88, 214.95)	< 0.01
TG (mg/dl)	99.12 (71.68, 138.95)	97.35 (70.8, 137.18)	109.74 (80.54, 151.34)	< 0.01
HDL-C (mg/dl)	51.03 (42.53, 61.08)	51.42 (42.91, 61.47)	48.71 (39.43, 59.83)	< 0.01
LDL-C (mg/dl)	114.05 (93.94, 135.31)	113.66 (93.17, 134.54)	119.07 (97.13, 140.63)	< 0.01
SBP (mm/Hg)	125 (113, 139.33)	124.33 (112.67, 138.67)	130 (118.33, 144.58)	< 0.01
DBP (mm/Hg)	74 (66.67, 82.33)	73.67 (66.33, 82)	76.33 (69.33, 85.33)	< 0.01
Non-HDLC	134.92 (112.89, 158.51)	134.15 (112.11, 157.73)	143.04 (120.23, 165.08)	< 0.01
TG/HDLC	1.93 (1.24, 3.07)	1.9 (1.22, 2.99)	2.29 (1.42, 3.6)	< 0.01
AI	2.66 (2.01, 3.48)	2.63 (1.99, 3.42)	3.01 (2.14, 3.86)	< 0.01
AIP	0.29 (0.09, 0.49)	0.28 (0.09, 0.48)	0.36 (0.15, 0.56)	< 0.01
LCI	41,471.98 (23,697.46, 73,052.40)	40,314.7 (23,195.04, 70,525.15)	52,735.08 (29,520.87, 91,568.91)	< 0.01

Chi-square test, rank-sum test and t-test were used to calculate *p*-values for categorical variables, continuous variables without normal distribution, and continuous variables with normal distribution, respectively

### Association and Dose–response relationship between five atherosclerosis indexes and T2DM

Table 2 showed the association of five atherosclerosis indexes with the risk of T2DM, as well as the quartiles of the five atherosclerosis indexes. After adjusting for above-mentioned covariates, results from multi-variate logistic regression showed that those five atherosclerosis indexes were related to the risk of T2DM when they were analyzed as continuous variables. Every IQR increase of Non-HDL-C meant 29% higher risk of T2DM (OR = 1.29; 95% CI = 1.15–1.45); every IQR increase of TG/HDLC meant 29% greater risk of T2DM (OR = 1.29; 95% CI = 1.18–1.42); every IQR increase of AI meant 45% greater risk of T2DM (OR = 1.45; 95% CI = 1.29–1.62); every IQR increase of AIP meant 41% greater risk of T2DM (OR = 1.41; 95% CI = 1.25–1.59); and every IQR increase of LCI meant 34% greater risk of T2DM (OR = 1.34; 95% CI = 1.23–1.48). Incident T2DM gradually increased as elevated quartiles of the five atherosclerosis indexes (*P* for trend < 0.001). The Q4 groups in Non-HDL-C, TG/HDLC, AI, AIP, and LCI showed a higher T2DM risk (OR = 1.83, 95% CI = 1.43–2.36; OR = 1.77, 95% CI = 1.39–2.26; OR = 1.81, 95% CI = 1.42–2.30; OR = 1.77, 95% CI = 1.39–2.26, OR = 2.01, 95% CI = 1.58–2.58, respectively) compared to the Q1 groups.

**Table 2:** Associations between the five atherosclerosis indexes and incident T2DM in the CHARLS

	Model1	*P*	Model 2	*P*	Model 3	*P*
Non-HDL-C per IQR	1.34[1.19,1.50]	< 0.001	1.33[1.18,1.49]	< 0.001	1.29[1.15,1.45]	< 0.001
Quartiles of Non-HDL-C						
Q1	Reference	–	Reference	–	Reference	–
Q2	1.24 [0.96, 1.62]	0.105	1.24 [0.95, 1.62]	0.109	1.22 [0.94, 1.59]	0.143
Q3	1.68 [1.31, 2.16]	< 0.001	1.69 [1.31, 2.18]	< 0.001	1.65 [1.28, 2.14]	< 0.001
Q4	1.95 [1.53, 2.50]	< 0.001	1.94 [1.51, 2.49]	< 0.001	1.83 [1.43, 2.36]	< 0.001
*p* for trend		< 0.001		< 0.001		< 0.001
TG/HDL-C per IQR	1.29[1.19,1.42]	< 0.001	1.33[1.22,1.45]	< 0.001	1.29[1.18,1.42]	< 0.001
Quartiles of TG/HDL-C		< 0.001		< 0.001		< 0.001
Q1	Reference	–	Reference	–	Reference	–
Q2	1.09 [0.84, 1.42]	0.507	1.11 [0.85, 1.44]	0.44	1.07 [0.82, 1.39]	0.608
Q3	1.42 [1.11, 1.82]	0.006	1.46 [1.14, 1.88]	0.003	1.37 [1.07, 1.77]	0.013
Q4	1.82 [1.44, 2.32]	< 0.001	1.92 [1.51, 2.45]	< 0.001	1.77 [1.39, 2.26]	< 0.001
*p* for trend		< 0.001		< 0.001		< 0.001
AI per IQR	1.47[1.32,1.65]	< 0.001	1.49[1.33,1.67]	< 0.001	1.45[1.29,1.62]	< 0.001
Quartiles of AI						
Q1	Reference	–	Reference	–	Reference	–
Q2	0.97 [0.75, 1.27]	0.845	0.98 [0.75, 1.27]	0.861	0.96 [0.73, 1.25]	0.741
Q3	1.34 [1.05, 1.72]	0.02	1.37 [1.07, 1.75]	0.014	1.30 [1.01, 1.67]	0.041
Q4	1.89 [1.49, 2.39]	< 0.001	1.93 [1.53, 2.46]	< 0.001	1.81 [1.42, 2.30]	< 0.001
* p* for trend		< 0.001		< 0.001		< 0.001
AIP per IQR	1.42[1.26,1.60]	< 0.001	1.46[1.30,1.65]	< 0.001	1.41[1.25.1.59]	< 0.001
Quartiles of AIP						
Q1	Reference	–	Reference	–	Reference	–
Q2	1.09 [0.84, 1.42]	0.507	1.11 [0.85, 1.44]	0.44	1.07 [0.82, 1.39]	0.608
Q3	1.42 [1.11, 1.82]	0.006	1.46 [1.14, 1.88]	0.003	1.37 [1.07, 1.77]	0.013
Q4	1.82 [1.44, 2.32]	< 0.001	1.92 [1.51, 2.45]	< 0.001	1.77 [1.39, 2.26]	< 0.001
* p* for trend		< 0.001		< 0.001		< 0.001
LCI per IQR	1.36[1.25,1.49]	< 0.001	1.38[1.26,1.51]	< 0.001	1.34[1.23,1.48]	
Quartiles of LCI						
Q1	Reference	–	Reference	–	Reference	–
Q2	1.14 [0.87, 1.48]	0.341	1.12 [0.86, 1.47]	0.393	1.11 [0.85, 1.45]	0.444
Q3	1.46 [1.14, 1.89]	0.003	1.48 [1.15, 1.92]	0.003	1.42 [1.10, 1.84]	0.007
Q4	2.12 [1.67, 2.70]	< 0.001	2.16 [1.70, 2.77]	< 0.001	2.01 [1.58, 2.58]	< 0.001
* p* for trend		< 0.001		< 0.001		< 0.001

Model 1 was rough model; Model 2 adjusted for age, gender, education, location and marriage; Model 3 further adjusted for smoking, drinking and sleep duration

IQR, interquartile range; AI, Index of atherogenicity; AIP, Atherogenic index of plasma; LCI, Lipid comprehensive index

**Fig. 1:**
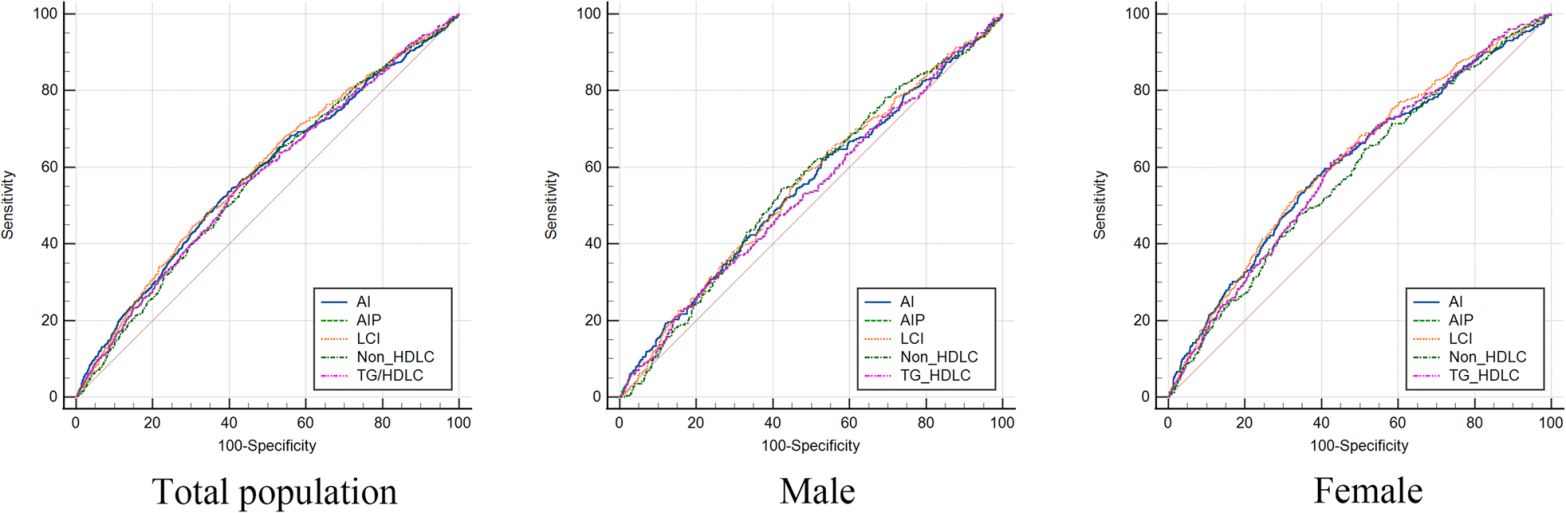
Receiver operating characteristic curves of five atherosclerosis indexes in total population, male and female for incident T2DM. The plot shows that LCI had the highest diagnostic value of all

Figure 1 showed the atherosclerosis index dose-dependent response of T2DM risk. A nonlinear relationship was found between the five atherosclerosis indexes and T2DM risk (*p* for nonlinear < 0.001) according to RCS, and higher levels of the atherosclerosis indexes gradually raised the overall risk of acquiring T2DM.

### Performance and predictive performance of the five atherosclerosis indexes to identify incident T2DM by gender

**Fig. 2:**
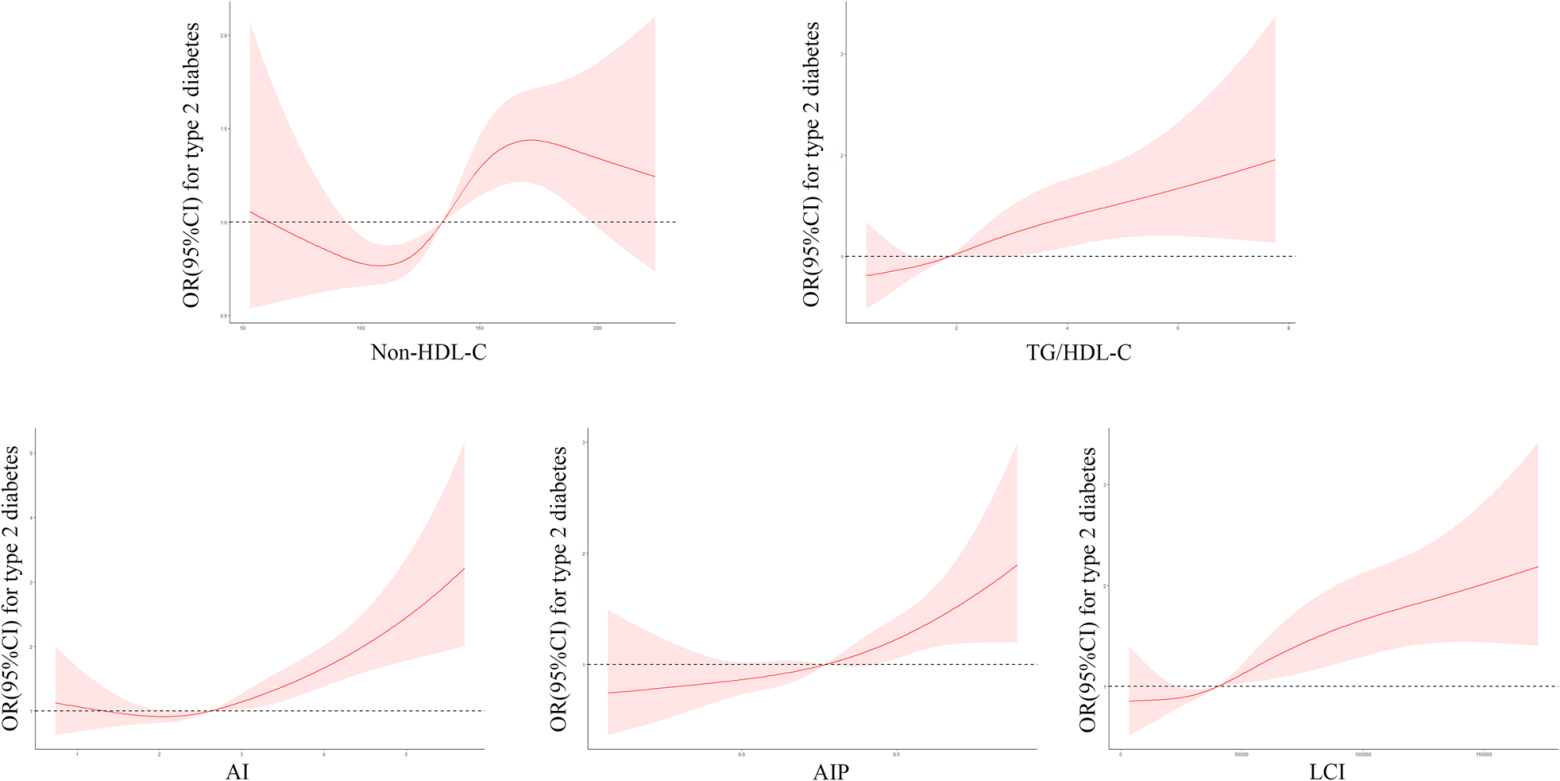
Adjusted cubic spline models of the association between five atherosclerosis indexes and risk of incident T2DM

Table 3 showed the predictive parameters of five atherosclerosis indexes for incident T2DM. Specially, LCI greatest the highest AUC (0.587 [0.574–0.600]), ranging from 0.568 to 0.579 in the study population, following by AI, TG/HDLC and AIP, Non-HDL-C. Non-HDL-C presented the highest sensitivity (0.605), while LCI presented the highest specificity (0.695). According to stratified analysis by gender, LCI presented higher diagnostic values than other indexes (Fig. 2). In female group, LCI also presented the highest AUC (0.618[0.600–0.635]), followed by AI (0.608[0.590, 0.626]), AIP (0.603[0.585,0.620]), TG/HDL-C (0.603[0.585–0.621]), and Non-HDL-C (0.582[0.564,0.600]); In male group, the predictive performance of all indexes were obviously lower than that in female group. Non-HDL-C and LCI presented the highest AUCs (0.553[0.533–0.572]; 0.553[0.534–0.572], respectively), followed by AI (0.547[0.528, 0.566]), TG/HDL-C (0.534[0.514,0.553]), and AIP (0.533[0.514–0.553]).

**Table 3:** Comparison of predictive accuracy and cut-off values of five atherosclerosis indexes by gender stratification

	Test	AUC	95CI Low	95CI Upp	Cutoff Value	Specificity	Sensitivity	PPV	NPV	*P*
Female	Non-HDL-C	0.582	0.564	0.6	151.933	66.04	47.59	0.151	0.908	< 0.001
	TG/HDL-C	0.603	0.585	0.621	2.1891	57.59	61.14	0.155	0.921	< 0.001
	AI	0.608	0.59	0.626	2.953	61.06	57.83	0.159	0.919	< 0.001
	AIP	0.603	0.585	0.62	0.340	57.59	61.14	0.155	0.921	< 0.001
	LCI	0.618	0.600	0.635	61,828.412	66.35	53.31	0.167	0.918	< 0.001
Male	Non-HDL-C	0.553	0.533	0.572	135.696	57.7	54.55	0.141	0.909	0.003
	TG/HDL-C	0.534	0.514	0.553	3.001	76.3	30.42	0.140	0.896	0.070
	AI	0.547	0.528	0.566	2.505	47.19	61.89	0.130	0.907	0.012
	AIP	0.533	0.514	0.553	0.477	76.25	30.42	0.140	0.896	0.071
	LCI	0.553	0.534	0.572	37,843.399	51.93	59.09	0.135	0.909	0.004
Overall	Non-HDL-C	0.568	0.554	0.581	135.696	52.2	60.52	0.138	0.912	< 0.001
	TG/HDL-C	0.571	0.557	0.584	2.14611	57.4	55.18	0.141	0.910	< 0.001
	AI	0.579	0.566	0.592	2.885	59.41	54.53	0.146	0.911	< 0.001
	AIP	0.57	0.557	0.584	0.3316	57.4	55.18	0.141	0.910	< 0.001
	LCI	0.587	0.574	0.6	61,830.459	69.49	44.82	0.157	0.908	< 0.001

### AUC difference between LCI and other four indexes by gender in the total sample

LCI and other atherosclerosis indexes for screening T2DM are shown in Table 4. We found that there were significant differences in AUC values between LCI and Non-HDL-C, TG/HDL-C, and AIP in overall population (P < 0.05), excluding AI. LCI might have higher predictive performance to identify T2DM compared to other surrogate indexes.

**Table 4:** Comparison of AUC values between LCI and other indexes

	Difference between area (95%CI)	*P*-value
Female		
LCI VS Non-HDL-C	0.035 [0.015, 0.055]	< 0.001
LCI VS TG/HDL-C	0.015 [− 0.0038, 0.033]	0.120
LCI VS AI	0.009 [− 0.004, 0.023]	0.189
LCI VS AIP	0.015 [− 0.003, 0.033]	0.118
Male		
LCI VS Non-HDL-C	0.000 [− 0.022, 0.022]	0.981
LCI VS TG/HDL-C	0.019 [− 0.000, 0.039]	0.057
LCI VS AI	0.006 [− 0.011, 0.023]	0.480
LCI VS AIP	0.019 [− 0.000, 0.039]	0.056
Overall		
LCI VS Non-HDL-C	0.019 [0.004, 0.034]	0.011
LCI VS TG/HDL-C	0.016 [0.002, 0.030]	0.020
LCI VS AI	0.007 [− 0.003, 0.018]	0.177
LCI VS AIP	0.016 [0.002, 0.030]	0.019

## Discussion

In this prospective cohort study, we analyzed the baseline and follow-up data of 4,596 eligible participants and found that the five atherosclerosis indexes were significantly associated with the risk of T2DM. Among the five indexes, LCI presented the highest predictive performance.

Dyslipidemia in diabetic patients has received a lot of attention in the past. It is prevalent in patients with T2DM and is believed to be the major cause of considerable CVD-related morbidity and mortality [14, 15]. And it is also well known that patients with diabetes and hyperlipidemia have a higher probability of adverse cardiovascular events and mortality [16]. Hyperglycemia is a very late stage in the chain of events from insulin resistance to diabetes, while lipoprotein abnormalities are mainly manifested in the asymptomatic diabetic prophase and contribute significantly to the increased risk of macrovascular disease [14, 17]. Recent studies have shown that T2DM is associated with increased hepatic and intestinal lipoprotein secretion, leading to the accumulation of triglyceride (TG)-rich lipoproteins in atherosclerosis. Large-scale clinical trials have proved the preventive effect of Atorvastatin on diabetes and metabolic syndrome [18].

Dyslipidemia often coexists with diabetes mellitus due to the close relationship between lipid and glucose metabolism. Insulin resistance is the primary cause of T2DM, and it also contributes significantly to hypertriglyceridemia. Therefore, the most common type of dyslipidemia in diabetic patients is hypertriglyceridemia [19, 20]. Domenico Trico et al. demonstrated that high triglyceride levels per se would deteriorate glucose tolerance and insulin sensitivity [21]. Studies showed that mild acute hypertriglyceridemia would impair glucose tolerance in healthy lean subjects by inducing insulin resistance, β cell dysfunction, and enhanced rate of oral glucose appearance, which were only partially compensated by the hyperglycemia-driven higher insulin secretion and β cell potentiation. In patients with dyslipidemia, this condition is more likely to be the first in the chain of events that eventually results in diabetes [5, 21, 22].

Previous studies suggested that plasma total cholesterol (TC), triglyceride (TG), and low-density lipoprotein cholesterol (LDL-C) were risk factors for coronary heart disease (CHD), while plasma high-density lipoprotein cholesterol (HDL-C) was a protective factor for CHD. And blood lipid disorders are usually cause by various degrees of changes of those indexes. Hu Lin believed that TC/HDL-C, LDL-C/HDL-C, and TC-HDL/HDL-C indexes were more useful in the diagnosis of CHD than single blood lipid index. LCI is a ratio calculated from the detection values of each blood lipid index, with a numerator of the risk factor for CHD and a denominator of the protective factor of CHD. The higher the LCI, the higher the exposure rate of risk factor, so it is scientific and credible to evaluate the risk of CHD [23]. Yin Boying et al. [24]believed that LCI, calculated using TC, TG, LDL-C, and HDL-C and presented higher sensitivity than any single index of blood lipids, could better reflect the effect of dyslipidemia on CHD. It is not reliable to evaluate and predict the risk of CHD by the changes of single blood lipid index [24]. Composite lipid indexes such as LCI and AIP are better means of predicting CAD risk than single lipid index [25]. And the related metabolic indexes, which are recognized as surrogates of insulin resistance, have been demonstrated to be relevant to clinical prognosis. Previous studies mostly focused on API and found that API was not only linked to CVD but could also predict the occurrence of T2DM [26]. Non-HDL-C is a new index linked to blood lipids after TG. Compared to LDL-C, the level of Non-HDL-C levels seems to be even more strongly linked to the development of coronary atheroma [27].

The blood lipids indexes presented excellent predictive performance not only on cardiovascular CVD but also on the occurrence of T2DM. These indexes were cost-effective and convenient, and could be detected and intervened in very early stage. In clinical settings, particularly in the department of cardiology, the detection of blood lipids has become a routine test, which has paved the way for practical application. Therefore, LCI has a good clinical application prospect for evaluating and predicting the occurrence of diseases. This study offered a possible route for anticipating T2DM risk. However, there are certain restrictions. First, most of our participants were not sure about whether they used lipid-lowering or hypoglycemic drugs, so we did not include this factor in the analysis. Second, this study's follow-up time was somewhat limited. As the bulk of the included participants were middle-aged and older people, more study on other age groups is required.

## Conclusions

This study demonstrated that LCI was a better predictor for T2DM identification in middle-aged and older Chinese in the clinical context, comparing to other indexes. LCI had the best sensitivity and the highest AUC value (0.587 [0.574–0.600]) among the five examined indexes. LCI might be a powerful and better independent predictor of T2DM risk compared to the traditional lipid indexes.

## Data Availability

Publicly available datasets were analyzed in this study. This data can be found at: China Health and Retirement Longitudinal Survey (2020). Available online at: http://charls.pku.edu.cn/pages/data/111/zh-cn.html.

## Abbreviations

DM: Diabetes mellitus
T2DM: Type 2 diabetes mellitus
LCI: Lipid comprehensive index
HDL-C: High-density lipoprotein cholesterol
TG: Triglyceride
TC: Total cholesterol
AI: Index of atherogenicity
AIP: Atherogenic index of plasma
BMI: Body mass index
CVD: Cardiovascular disease
ASCVD: Atherosclerotic cardiovascular disease
WC: Waist circumference
DBP: Diastolic blood pressure
SBP: Systolic blood pressure
